# Overexpression of HMGB1 in melanoma predicts patient survival and suppression of HMGB1 induces cell cycle arrest and senescence in association with p21 (Waf1/Cip1) up-regulation via a p53-independent, Sp1-dependent pathway

**DOI:** 10.18632/oncotarget.2201

**Published:** 2014-07-23

**Authors:** Qingling Li, Jie Li, Ting Wen, Weiqi Zeng, Cong Peng, Siyu Yan, Jieqiong Tan, Keda Yang, Shuang Liu, Aiyuan Guo, Chong Zhang, Juan Su, Minghao Jiang, Zhaoqian Liu, Honghao Zhou, Xiang Chen

**Affiliations:** ^1^ Department of Dermatology, Xiangya Hospital, Central South University, Changsha, 410008, China; ^2^ Department of Orthopaedics, Xiangya Hospital, Central South University, Changsha, 410008, China; ^3^ State Key Laboratory of Medical Genetics, Xiangya Medical School, Central South University, Changsha, 410008, China; ^4^ Department of Pathology, Xiangya Hospital, Central South University, Changsha, 410008, China; ^5^ Department of Anesthesiology, Xiangya Hospital, Central South University, Changsha, 410013, China; ^6^ Institute of Clinical Pharmacology, Xiangya School of Medicine, Central South University, Changsha, 410008, China

**Keywords:** high mobility group box 1, p53, p21, Sp1, melanoma

## Abstract

Although laboratory studies have implicated the high mobility group box 1 (HMGB1) in melanoma, its clinical relevance remains unclear. We analyzed nearly 100 cases of human melanoma and found that HMGB1 was highly overexpressed in melanoma samples relative to normal skin and nevi tissues. Significantly, higher levels of HMGB1 correlated with more advanced disease stages and with poorer survival in melanoma patients. Unlike the well-documented pro-inflammatory role of the extracellular HMGB1, we found that its intracellular activity is necessary for melanoma cell proliferation. An absolute dependency of melanoma cell proliferation on HMGB1 was underscored by the marked response of cell cycle arrest and senescence to HMGB1 knockdown. We demonstrated that HMGB1 deficiency-induced inhibition of cell proliferation was mediated by p21, which was induced via a Sp1-dependent mechanism. Taken together, our data demonstrate a novel oncogenic role of HMGB1 in promoting human melanoma cell proliferation and have important implications in melanoma patient care.

## INTRODUCTION

The high mobility group box 1 (HMGB1) protein, a DNA-binding protein with many additional functions, is ubiquitously present in the nucleus of almost all mammalian cells. HMGB1 is composed of three domains, two basic HMG boxes domain (HMG boxes A and B) and a highly acidic C-terminal domain. In the nucleus, HMGB1 has been shown to be involved in a variety of biologically important processes, including nucleosomes stabilization, transcriptional regulation, V(D)J recombination, DNA repair and genomic stability [1, 2]. HMG boxes A and B facilitate transcriptional protein with a binding to specific DNA target for the characteristic structure of L-shaped fold [3], while the acidic C-tail is involved in transcriptional stimulation [4] and inhibition [5]. What's more, HMGB1 can also be subjected to post-translational modification which can fine-tune interactions of the proteins with DNA/chromatin and determine their relocation from the nucleus to the cytoplasm and secretion [3]. In addition to its nuclear role, HMGB1 is actively secreted by immune cells or passively released from necrotic cells, in response to injury and inflammatory stimuli.

Although HMGB1 overexpression is observed in a number of malignancies, its role in cancer has not been fully elucidated. And also HMGB1 may support tumor growth and metastasis though its ability as an extracellular ligand and/or its pro-inflammatory properties as a damage-associated molecular pattern to induce proliferation or angiogenesis [2, 6-9]. Moreover, HMGB1-RAGE interaction conveys poor prognosis in colorectal carcinoma and prostate cancer [2]. However, because most studies have focused on the extracellular functions of HMGB1, its role, as an oncogene in tumor progression is largely unknown. The limited studies available suggest that HMGB1 may contribute to tumorigenesis by inhibiting apoptosis or regulating autophagy in transformed cells [10-12]. Although HMGB1 enhances promoter activity in melanoma cells [13], the functional role of HMGB1 in tumor progression and its association with clinical outcome of cancer patients is still not fully understood.

p21/WAF1/CIP1 is a universal cyclin-dependent kinase inhibitor (CDKI) that is associated with several CDK complexes to regulate the progression of cells through the cell cycle [14]. Previous studies have shown that in both normal [15] and tumor cell lines [16], the introduction of p21 expression constructs result in a cell cycle arrest at the G1 phase [17, 18]; p21 has an effective inhibitory capacity in the G1/S transition CDKs including CDK2, CDK3, CDK4, and CDK6 [18]. In addition, p21 has been shown possessing several important physiologic functions, such as apoptosis, differentiation, and senescence. Several tumor suppressors and oncogenes treat it as a downstream target and regulate its expression at the transcriptional or post-translational levels [19].

p21 was first found to be directly induced by p53 and mediate the p53-dependent cell cycle regulation of G1 phase arrest to control cell proliferation and cancer [16]. In addition to p53, numerous studies have reported that Sp1 is another essential factor in the regulation of p21 [20-23]. Sp1 is a member of a multigene family that binds DNA through C-terminal zinc-finger motifs [24, 25]. In the promoter of p21, there are six Sp1 binding sites (Sp1-1 to Sp1-6) playing an important role in p21 transcriptional regulation. In response to phorbol ester (PMA) and okadaic acid, an induction of the p21 gene is mediated by Sp1 through Sp1-1 and Sp1-2 sites in human U937 cells undergoing differentiation toward macrophages [26]. Moreover, Sp1 can also coordinate with p300/CBP for an induction of p21 [27].

Although substantial information regarding the role of HMGB1 in inflammation exists, HMGB1 is essentially uncharacterized as an oncoprotein in tumorigenesis. Here, we reported for the first time that high levels of HMGB1 in melanoma correlate with advanced disease stages and poor patient survival. We showed that HMGB1 is crucial in melanoma cell proliferation because HMGB1 depletion leads to a dramatic inhibition of melanoma cell proliferation both *in vivo* and *in vitro*. Moreover, the targeted ablation of HMGB1 results in p21-dependent cell cycle arrest and senescence. Interestingly, the p21 transaction is Sp1-dependent. Additionally, the results obtained through chromatin immunoprecipitation assays and co-immunoprecipitation assays suggested a co-binding of HMGB1 and Sp1 in p21 promoter for its transcriptional regulation. These findings define a new mechanism for HMGB1 in promoting tumor growth by enhancing proliferation and preventing senescence in melanoma cells.

## RESULTS

### Increased expression of HMGB1 is correlated with the progression of human cutaneous melanoma and poor patient survival

**Figure 1:**
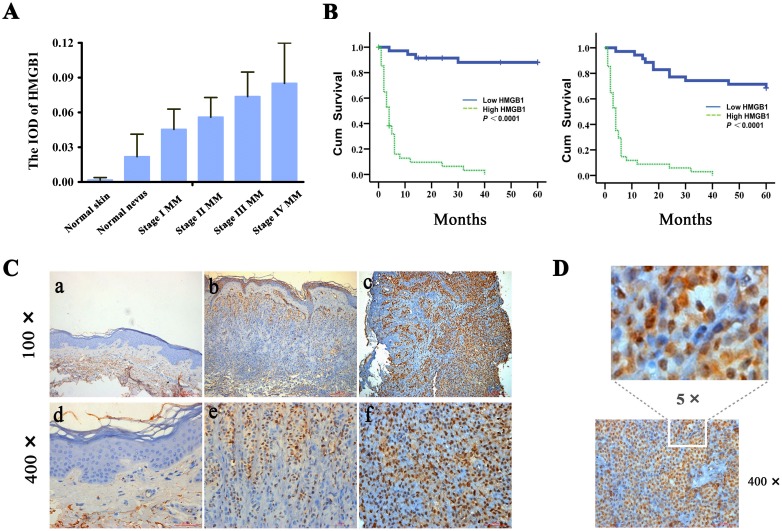
Increased expression of HMGB1 is correlated with the progression of human cutaneous melanoma and poor patient survival **(A)** The expression levels of HMGB1 (IOD) in normal skin, normal nevus, and various melanoma stages (stage I, stage II, stage III and stage IV). TNM classification and clinical stage are according to the melanoma staging system of the American Joint Committee on Cancer (AJCC). The mean IOD of HMGB1 in each group is shown in a bar figure and presented as mean ± SD. As shown, melanomas have increased expression of HMGB1 compared with normal skin and nevi. Low-grade tumors have lower average levels of expression compared with high-grade tumors. Bars: SD; p<0.0001. IOD, integral optical density; MM, malignant melanoma. **(B)** Kaplan-Meier survival curves for groups based on HMGB1. HMGB1 staining is correlated with overall 5-year survival (left panel), and disease-specific 5-year survival (right panel) (both p<0.0001; log-rank test). Cum,cumulative. **(C)** Representative immunohistochemical analysis of HMGB1 expression in normal skin (a and d), normal nevi (b and e),and melanoma (c and f). (D) High magnification of HMGB1 detected in melanoma tissue section. As shown, HMGB1 protein was localized mainly inside the melanoma cells.

A potential contribution of HMGB1 to melanoma has been suggested from preclinical studies [13], but awaits further validation with the clinical evidence. We addressed this need by analyzing the expression of HMGB1 using immunohistochemistry in 102 cases of human melanoma. When compared with normal skin and nevi tissues, there was a significantly elevated expression of HMGB1 detected in the majority of the melanoma specimens (Figure 1A). To gain a better understanding of this association, we examined the correlation of the HMGB1 level and the melanoma stage. According to the American Jiont Committee on Cancer (AJCC) melanoma staging criteria [28, 29], the melanoma patients were grouped into stage I, II, III and IV. Analysis of the expression data with the melanoma stage uncovered a clear positive correlation between the disease stage and the level of HMGB1 expression, as reflected by markedly higher HMGB1 protein levels detected in the more advanced stages of melanoma. In line with such an association, a number of clinicopathological characteristics of higher-grade melanoma including the tumor thickness, mitotic index, lymph node metastasis, and distance metastasis also exhibited a positive correlation with a higher level of HMGB1 (Table 1). This close association of a high level of HMGB1 with the late disease stage prompted us to examine its relationship with the melanoma patient prognosis. The Kaplan-Meier curve of HMGB1 levels and patient outcomes revealed a significant correlation of the level of HMGB1 expression and melanoma patient survival. Higher HMGB1 levels are associated with significantly poorer overall and melanoma-specific 5-year survival (p<0.001) (Figure 1B). Our data together provide much needed clinical evidence supporting an oncogenic role of HMGB1 in human melanoma. Our study also implicates HMGB1 as a new molecular prognostic marker for this aggressive disease.

**Table 1:** Association between HMGB1 expression and clinicopathologic characteristics of melanomas

Variable	HMGB1 staining			p value^*^
	Low	High	Total	
Age, years^**^				0.69
≤ 59	27(51.9%)	25(48.1%)	52	
> 59	24(48.0%)	26(52.0%)	50	
Sex				0.42
Male	28(46.7%)	32(53.3%)	60	
Female	23(54.8%)	19(45.2%)	42	
**Tumor thickness, mm**				0.012
≤ 2.0	23(67.6%)	11(32.4%)	34	
>2.0	28(41.2%)	40(58.8%)	68	
**Ulceration**				0.29
Present	32(46.4%)	37(53.6%)	69	
Absent	19(57.6%)	14(42.4%)	33	
**American Joint Committee on Cancer stage**				<0.0001
I	12(92.3%)	1(7.7%)	13	
II	28(82.4%)	6(17.6%)	34	
III	6(18.7%)	26(81.3%)	32	
IV	3(21.4%)	11(78.6%)	14	
**Tumor subtype**				0.75
Superficial spreading melanoma	21(46.7%)	24(53.3%)	45	
Lentigo maligna melanoma	3(75.0%)	1(25.0%)	4	
Acrolentigous melanoma	11(55.0%)	9(45.0%)	20	
Nodular melanoma	8(61.5%)	5(38.5%)	13	
Unspecified	6(54.5%)	5(45.5%)	11	
**Mitotic index**				<0.0001
≤0.75	44(72.1%)	17(27.9%)	61	
>0.75	7(17.1%)	34(82.9%)	41	
**Tumor location**				0.505
Sun-exposed (head and neck)	4(40.0%)	6(60.0%)	10	
Sun-protected (others)	47(51.1%)	45(48.9%)	92	
**Lymph node metastasis**				<0.0001
Present	9(19.6%)	37(80.4%)	46	
Absent	40(85.1%)	7(14.9%)	47	
**Distant metastasis**				0.011
Present	3(21.4%)	11(78.6%)	14	
Absent	46(58.2%)	33(41.8%)	79	

* Analysis by *χ^2^* test or *Fisher's* exact test;

** For the 102 melanoma cases, the median age of the whole group of patients was 59 years (range, 17–89 years).

Available information indicates that the HMGB1 protein has diverse biological functions ranging from inflammation, genome stability, proliferation, cell death, autophagy, to tumor metastasis [1, 2, 6]. To further explore the connection between HMGB1 with the progression of human melanoma, we examined the status of cell proliferation by measuring the mitotic index. In addition to that mitotic index showed a positive correlation with a higher level of HMGB1 (Table 1), a multivariate analysis including mitotic index, HMGB1 expression, age, sex, thickness, ulceration, AJCC stage, and location of the primary melanomas indicates that it is the most significant prognostic marker for disease-specific 5-year survival (relative risk=17.36, 95% CI=1.72 to 174.83; p=0.015; Table 2). Like AJCC stage, which has been widely accepted as an independent prognostic factor for melanoma patient survival, HMGB1 expression is an independent prognostic factor for overall (relative risk=6.14, 95% CI=2.25 to 16.76; p<0.0001, Table 2) and disease-specific 5-year survival (relative risk=3.81, 95% CI=1.15 to 12.59; p=0.028; Table 2). Our data are consistent with the result from a previous study [30] showing that the mitotic index also correlated with the disease stage, i.e., a higher mitotic index can be found in a more advanced stage of melanoma. Taken together, these data suggest a tight association between melanoma with an elevated rate of cell proliferation with increased HMGB1 expression.

**Table 2:** Multivariate Cox regression analysis of HMGB1 overexpression with disease-specific 5-year and overall survival in 70 cases of primary melanomas

Variable	Disease-Specific Survival			Overall Survival		
	Relative Risk	95% CI	p value	Relative Risk	95% CI	p value
HMGB1	3.81	1.15 -12.59	0.028	6.14	2.25-16.76	<0.0001
Mitotic index	17.36	1.72-174.83	0.015	-	-	0.371
AJCC stage	3.78	1.17-12.26	0.027	4.97	1.84-13.48	0.002

Coding of variables: HMGB1 was coded as 1, low expression; and 2, high expression. Thickness was coded as 1, ≤2 mm; and 2, >2 mm. Ulceration was coded as 1, absent; and 2, present. Location was coded as 1, extremities and trunk; and 2, head and neck. Age was coded as 1, ≤ 59 years; and 2, >59 years. Sex was coded as 1, male; and 2, female; Mitotic index was coded as 1, ≤0.75; and 2, >0.75; AJCC stage was coded as 1, stage I, II; and 2, stage III.

Accumulating evidence indicate that HMGB1 can function either inside or outside of a cell, and the extracellular HMGB1 plays an important role in inflammation that contributes to the development of cancer [6-9, 11]. We sought to link the distribution of the HMGB1 protein to its oncogenic activity observed in human melanoma. A close examination of melanoma patient samples discovered that the HMGB1 protein was mainly localized inside the cancer cells (Figure 1C and D), suggesting an activity mediated by the intracellular HMGB1 at work in human melanoma. Taken together, our data implicate an oncogenic function of the intracellular HMGB1, which is associated with an increased cell proliferation in human melanoma tissues.

### Downregulation of HMGB1 in melanoma cells induced inhibition of cell proliferation, cell cycle arrest and senescence

**Figure 2:**
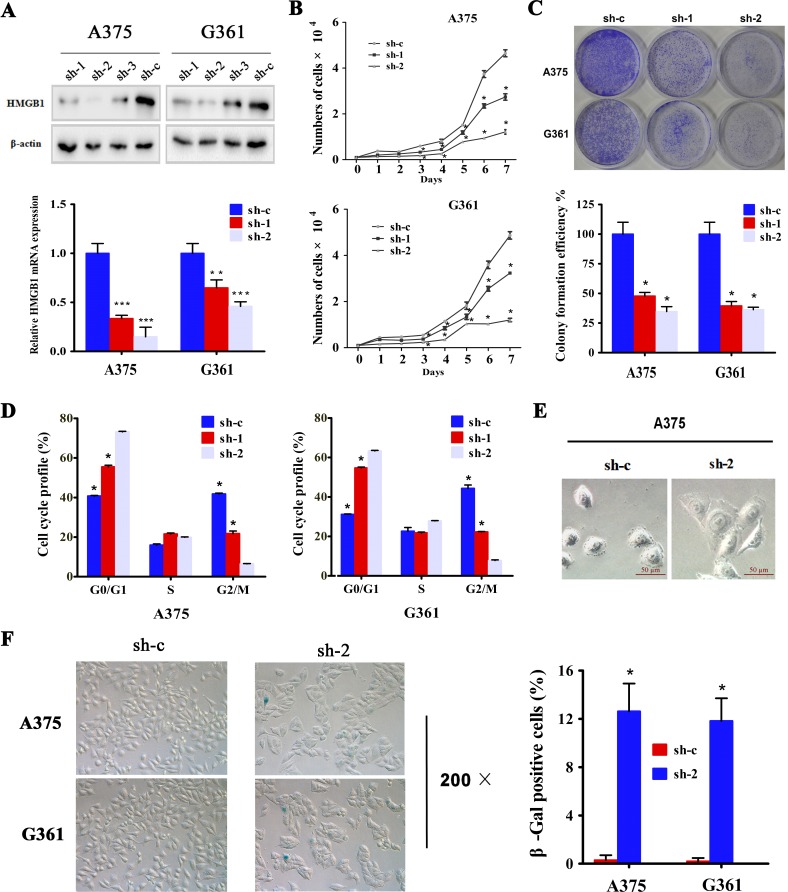
Downregulation of HMGB1 in melanoma cells induced inhibition of cell proliferation, cell cycle arrest and senescence **(A)** A375 and G361 cells were transfected with three independent shRNA constructs against HMGB1 (sh-1, sh-2 and sh-3) or control shRNA (sh-c). The protein levels of HMGB1 were analysis by immunoblot (upper panel), and the mRNA levels of HMGB1 were analysis by qRT-PCR (lower panel). Bars, SD; **p<0.005,***p<0.0005. (**B**) Growth curve of A375 or G361 expressing shRNA HMGB1 (sh-1 or sh-2) or the control shRNA (sh-c) was determined. The numbers were mean ± SD from three experiments performed in triplicate. Bars, SD; *p<0.05. (**C**) Cells as in B were subjected to colony formation assay. Representative colony images of A375 and G361 are shown (upper panel). The bar chart shows a quantitative analysis of the colony formation assay (lower panel). The numbers were mean ± SD from three experiments performed in triplicate. Bars, SD; *p<0.05. (**D**) A375 and G361 expressing shRNA targeting HMGB1 (sh-1 or sh-2) or control (sh-c) were labeled with BrdU 30 min before harvesting and cell cycle distribution was analyzed with FACS analysis. The proportions of cells in each cell cycle were counted and presented as mean ± SD from three independent experiments. Bars, SD; *p<0.05. (**E**) Representative morphology of HMGB1 depleted (sh-2) or control (sh-c) A375 cells are shown. (**F**) A375 (upper panel) and G361 (lower panel) expressing HMGB1 shRNA (sh-2) or control shRNA (sh-c) were analyzed by SA-β-gal staining. The representative phase images are shown. The percentage of SA-β-gal positive cells were counted and presented as mean ± SD from three independent experiments performed in triplicate. Bars, SD; *p<0.05.

The association of HMGB1 protein abundance with highly elevated mitotic index from melanoma patient samples led us to ask whether this high mobility protein might contribute to melanoma cell proliferation. We addressed this question by employing shRNAi-mediated knockdown to deplete the expression of HMGB1 (Figure 2A). To avoid the potential off-target effect, we used multiple shRNAi sequences to target the HMGB1 gene and complemented it with re-introduction of a HMGB1 encoding plasmid to the HMGB1-deficient cells (Supplementary Figure S1A). We took the advantage of the different levels of knockdown by the sequence #1 and #2 (sh-1 & sh-2) for assessing a dosage-dependent contribution of HMGB1 to melanoma cell proliferation. Measurement of cell growth curve revealed a critical role of HMGB1 in supporting melanoma cell proliferation. Knockdown of HMGB1 expression resulted in a marked reduction of cell proliferation and importantly, this effect appeared to be nicely correlated with the level of HMGB1 expression (Figure 2B). The retarded rate of cell proliferation in the shRNAi expressing cells was completed rescued upon re-expression of HMGB1 (Supplementary Figure S1B), confirming that HMGB1 is responsible for the control of cell proliferation. In addition, two different melanoma cell lines exhibited a similar response to reduced HMBG1 expression, indicating that the role of HMGB1 in melanoma cell proliferation is not cell line-dependent. To substantiate the cell growth curve data, we carried out the colony formation assay. The results again indicated a dose-dependent effect of HMGB1 in regulation of melanoma cell proliferation, higher levels of HMGB1 expression correlated with high rates of cell proliferation (Figure 2C). These data are consistent with the observations that higher HMGB1 expression was associated with greater mitotic index in human melanoma patient samples, supporting a critical role of HMGB1 in supporting melanoma cell proliferation.

To gain a better understanding of how HMGB1 contributes to melanoma cell proliferation, we examined cell cycle progression. Relative to the control cells, HMGB1-deficient cells showed a significant delay in cell cycle progression (Figure 2D). Quantification of cell population at each cycle phase revealed a clear accumulation of cells in the G1 phase, indicative of a G1 cell cycle arrest. While observing cell morphology, we noticed that the HMGB1 stable knockdown cells appeared to be flatter in shape and larger in size than the matched control cells (Figure 2E). This morphology change prompted us to ask whether HMGB1 depletion might induce cellular senescence. Measurement of senescent-associated β-galactosidase activity showed a marked increase of β-Gal-positive cells (Figure 2F), confirming an induction of cellular senescence by HMGB1 depletion. The induction of cell cycle arrest and senescence is consistent with the decreased rate of cell proliferation in HMGB1-knockdown melanoma cells. In parallel, analysis of cell cycle by PI staining yielded similar results (Supplementary Figure S1C-E).

### HMGB1 depletion was associated with p21 upregulation in a p53 independent manner

**Figure 3:**
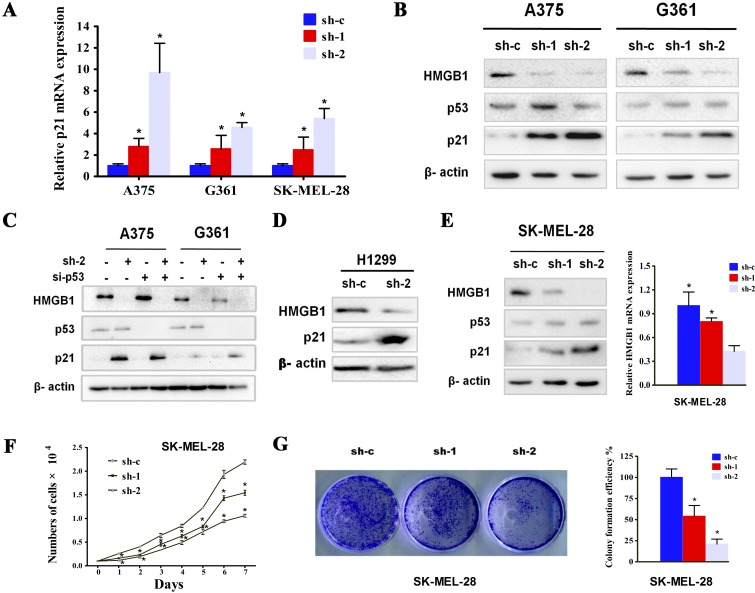
HMGB1 depletion was associated with p21 upregulation in a p53 independent manner **(A)** The mRNA levels of p21 from A375, G361 or SK-MEL-28 cell line expressing the indicated shRNA were determined by qRT-PCR. The numbers were mean ± SD from three experiments performed in triplicate. Bars, SD; *p<0.05. (**B**) The cells as in A were subjected to Western blot analysis using the indicated antibodies. (**C**) A375 and G361 cells were transfected with p53 siRNA (si-p53) or control siRNA (si-c), with or without stable HMGB1 depletion. The cells were analyzed via Western blot using the indicated antibodies. (**D**) H1299 (p53-null) cells were transfected with either control (sh-c) or HMGB1 (sh-2) shRNA and the cells were analyzed for the expression of HMGB1 and p21. (**E**) The expression of HMGB1 was knocked down in SK-MEL-28 cells and the protein (left panel) and mRNA levels (right panel) were analyzed. Representative results from one of three independent experiments performed in triplicate are shown. Bars, SD; *p<0.05. (**F**) Growth curve of SK-MEL-28 expressing shRNA HMGB1 (sh-1 or sh-2) or the control shRNA (sh-c) was determined. The numbers were mean ± SD from three experiments performed in triplicate. Bars, SD; *p<0.05. (**G**) Cells as in F were subjected to colony formation assay. Representative colony images are shown (left panel). The bar chart shows a quantitative analysis of the colony formation assay (right panel). The numbers were mean ± SD from three experiments performed in triplicate. Bars, SD; *p<0.05.

Cell cycle progression depends on the activity of cyclin-dependent kinases (CDK), which are under the control by the CDK inhibitors. We asked whether HMGB1-deficiency induced cell cycle arrest was mediated by the CDK inhibitors. Indeed, measurement of a panel of CDK inhibitors' transcript indicated an increase in p21 specifically in HMGB1 shRNAi but not control shRNAi expressing cells (Figure 3A). The induction of p21 was also confirmed by immunoblotting (Figure 3B). As a critical inhibitor of G1 cell cycle kinase, the expression of p21 can be regulated by either p53-dependent or independent manner [31-33]. The p53 abundance however, was not detectably affected when HMGB1-depleted cells were compared with the control cells, suggesting a p53-independent mechanism of p21 regulation. We further tested the role of p53 by knocking down its expression. A comparable level of p21 induction by HMGB1 depletion was seen in both p53 proficient and deficient cells (Figure 3C), confirming that p53 is dispensable for the increase of p21 expression. In further support of this p53-independent induction of p21, knockdown of HMGB1 in p53 mutant expressing SK-MEL-28 melanoma cells and p53-null cell line, H1299, was also associated with a marked increase in p21 expression (Figure 3D and 3E). In addition, knockdown of HMGB1 expression in SK-MEL-28 cells also resulted in a marked reduction of cell proliferation (Figure 3F and 3G). Taken together, our data demonstrate a p53-independent mechanism of p21 induction in response to reduced expression of HMGB1.

### p21 is required for induction of cell cycle arrest and senescence upon HMGB1 depletion

**Figure 4:**
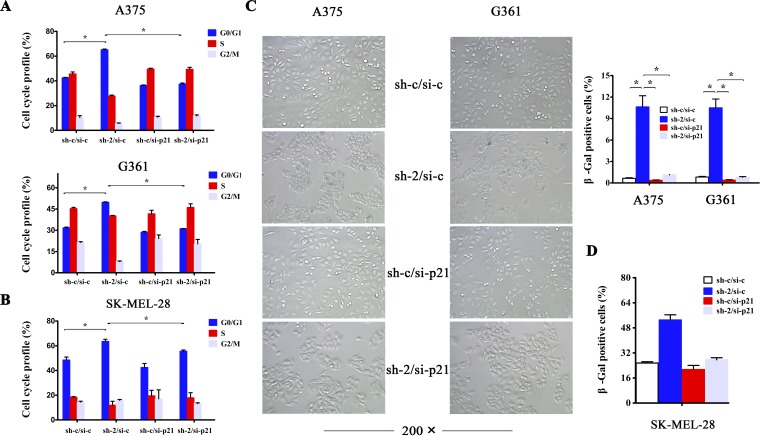
p21 is required for induction of cell cycle arrest and senescence upon HMGB1 depletion (**A**) A375 and G361 cells were transfected with the indicated sh or siRNA. The cells were analyzed with FACS for cell cycle distribution. The bar chart shows the percentage of cell population in A375 (upper panel) and G361 (lower panel) cell cycle. The numbers are mean ± SD from three independent experiments. Bars, SD; *p<0.05. (**B**) The same treatment as A was performed in SK-MEL-28 cells,and the bar chart shows the percentage of cell population in its cell cycle. The numbers are mean ± SD from three independent experiments. Bars, SD; *p<0.05. (**C**) A375 and G361 cells as in A were analyzed for senescent phenotype via SA-β-gal staining. Representative images were shown. SA-β-gal-positive cells were counted and the numbers represent mean values of three independent experiments. Bars, SD; *p<0.05. (**D**) The same treatment as A was performed in SK-MEL-28 cells,and SA-β-gal-positive cells were counted and the numbers represent mean values of three independent experiments. Bars, SD; *p <0.05.

We next investigated whether p21 was responsible for HMGB1-downregulation induced cell cycle arrest and senescence. We addressed this using RNAi to knockdown the expression of p21. The result indicates that depletion of p21 expression almost completely abrogated G1 cell cycle arrest induced by HMGB1 deficiency (Figure 4A and 4B). In addition, knockdown of p21 expression also abolished the senescent phenotype in HMGB1 deficient cells (Figure 4C and 4D). The data together demonstrate that HMGB1 depletion-induced cell cycle arrest and senescence is largely mediated by p21.

### Upregulation of p21 induced by HMGB1 knockdown is Sp1 dependent

**Figure 5:**
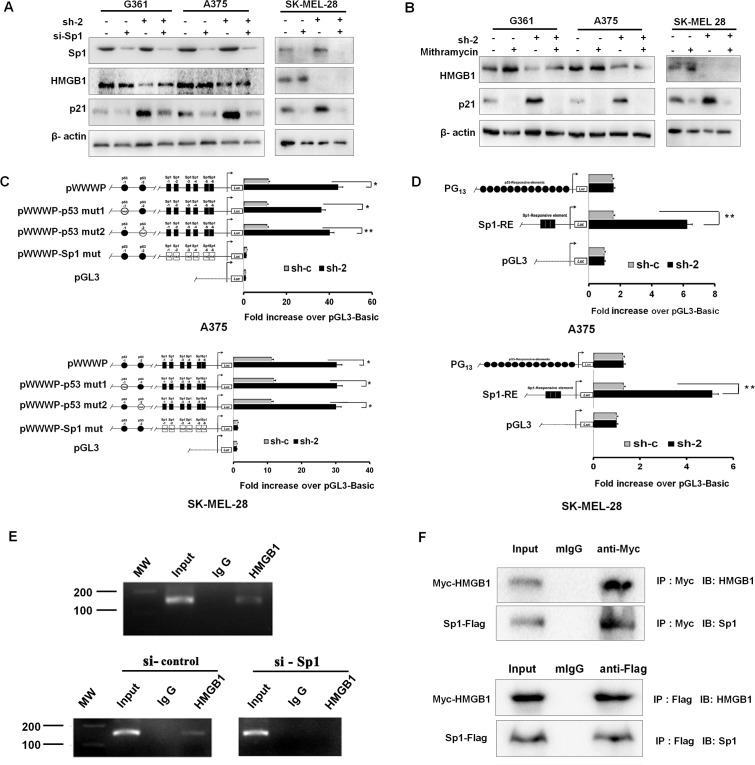
Upregulation of p21 induced by HMGB1 knockdown is Sp1 dependent (**A**) Control (sh-c) or HMGB1 knockdown (sh-2) A375, G361, and SK-MEL-28 cells were transfected with Sp1 siRNA (si-Sp1) or control siRNA (si-c). The cells were harvested for Western analysis of p21, HMGB1 and Sp1 expression. (**B**) Control or HMGB1 shRNA (sh-2) expressing A375, G361 and SK-MEL-28 cells were treated with mithramycin A (200 nM for 20 h) or control vehicle (DMSO), as indicated. The cells were harvested for Western analysis using the indicated antibodies. (**C**) Luciferase plasmids driving by wild-type or mutant (with p53-binding site mutated) p21 promoter were transfected into control (sh-c) or HMGB1 shRNA (sh-2) A375 (upper panel) and SK-MEL-28 (lower panel) cells. The luciferase assay was carried out 48 h post-transfection. The numbers are mean ± SD from three independent experiments. Bars, SD; *P<0.01 and **P<0.0001. (**D**) Similar luciferase assays but with Sp1-RE-Luc and PG13-Luc promoter plasmids were performed in A375 (upper panel) and SK-MEL-28 (lower panel). The results of three independent experiments performed in triplicate are shown. Values are mean of the ratio of Firefly luciferase activity to Renilla luciferase activity and then normalized to pGL3 vector activity. Bars, SD; *P<0.01 and **P<0.0001. (**E**) ChIP assays. The chromatins, which prepared from A375 cells (upper panel) and A375 cells transfected or not with si-control /si-Sp1 (lower panel), were performed with control IgG or anti-HMGB1 antibodies. The region of Sp1 binding site (spanning from −180 to −29) in the p21 promoter was amplified by PCR. (**F**) Plasmids expressing Myc-HMGB1 and Sp1-Flag were co-transfected into 293FT cells. Cells were harvested at 36h, and protein samples were prepared for IP with anti-Myc or anti-Flag and IgG as a control. Total cell lysates and immunoprecipitates were analyzed by Western Blots.

Having observed that HMGB1 knockdown was associated with p53-indendent upregulation of p21, we asked whether Sp1, a major p53-independent regulator of p21 [20-23], might be involved. We tested this by knockdown the expression of HMGB1 using RNAi. Remarkably, Sp1 silencing significantly attenuated the increase in p21 protein expression that was induced by HMGB1 knockdown in A375, G361, and SK-MEL-28 cells (Figure 5A). Furthermore, Mithramycin A, a highly specific inhibitor of Sp1 binding [34], also blocked the induction of p21 in HMGB1 deficient cells (Figure 5B), corroborating the results derived from knockdown experiments.

Sp1 is known to directly bind to the promoter and induce the expression of p21 [35, 36]. To further validate that Sp1 was responsible for the p21 induction in HMGB1 knockdown cells, we mutated the Sp1 binding site within the p21 promoter, with 2 mutants defective in p53-binding included as controls. Consistent with a Sp1-dependent and p53-independent mechanism of p21 regulation, mutation of Sp1 but not p53 binding sites abrogated p21 induction by HMGB1-deficience (Figure 5C and 5D). Almost identical results were obtained in both A375 and SK-MEL-28 cell lines. These data together confirm a Sp1-depdent mechanism of p21 induction by HMGB1-depletion.

In order to investigate how HMGB1 interacted with Sp1 in the transcriptional regulation of p21, we conducted a ChIP assay. As shown in Figure 5E, there was a binding of HMGB1 to the region of Sp1 binding site in p21 promoter in A375 cells, while silencing Sp1 disrupted the binding in p21 promoter. An additional support for this Sp1-mediated p21 regulation was the finding that HMGB1 and Sp1 directly associated with each other (Figure 5F). Taken together, these data implicate that HMGB1 interacts with Sp1 for the co-binding in p21 promoter and interferes with Sp1-mediated transcription of p21. Knockdown of HMGB1 results in alleviating its inhibitory effect leading to up-regulation of p21 transcription by Sp1.

### Association of HMGB1 with p21 and p53 in melanoma tissues

**Figure 6:**
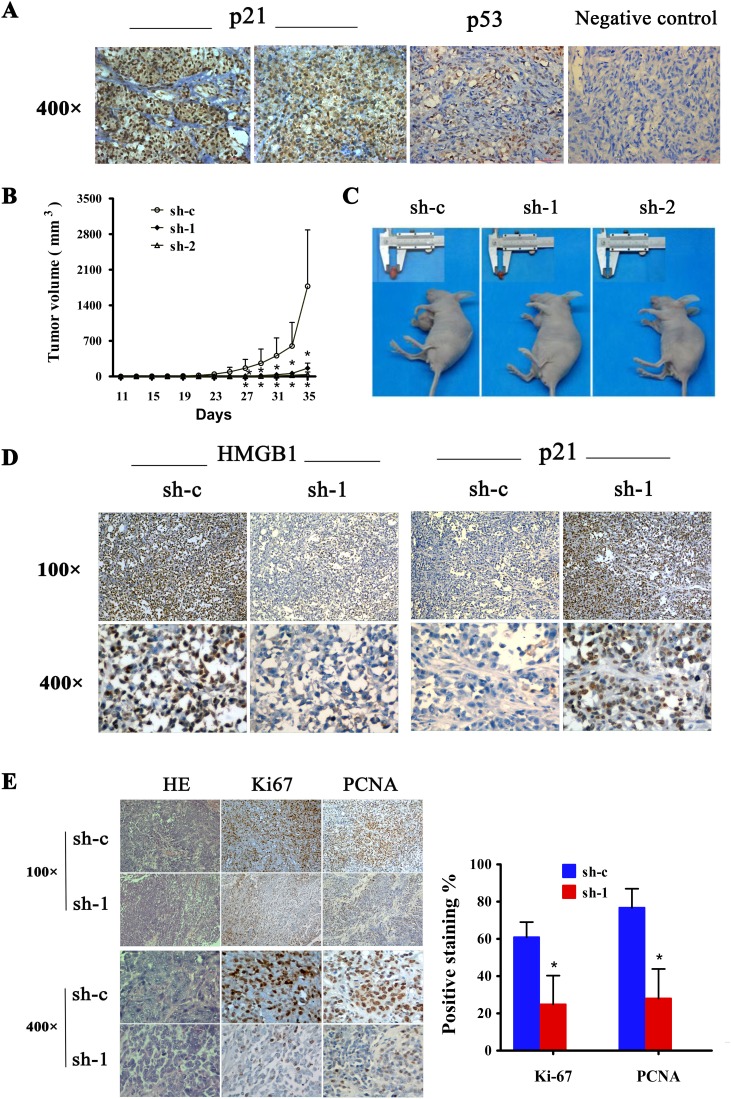
Expression of p21 and p53 in human cutaneous melanoma and effect of HMGB1 inhibition on tumorigenicy of melanoma cell lines (**A**) Representative immunohistochemical staining of p21 and p53 expression in melanoma tissues. MM,malignant melanoma. (**B**) The A375 cells (5×10^6^/0.2ml) expressing control (sh-c) or HMGB1 shRNA (sh-1 or sh-2) were implanted the flank of nude mouse. Tumor development was monitored 3 times /week. The numbers are means (n=6) of tumor volume ± SD. Bars, SD; *p=0.039. (**C**) Representative tumor sizes isolated from indicated mice at day 35 post-transplant. (**D**) Tumors were collected at day 35 post-transplantation. The tumor tissues were processed for immunohistochemical analysis. Representative images of immunohistochemical stain of tumor tissue sections with anti-HMGB1 or p21 antibodies isolated from mice transplanted with melanoma cells expressing sh-c and sh-1. (**E**) The tumor sections were also stained for H&E, Ki-67 or PCNA. The Ki-67 or PCNA positive counted and the numbers are means from sh-c and sh-1 group. Bars, SD; *p<0.0001.

To test the data derived from in vitro studies for the clinical relevance, we examined whether there was any association between HMGB1 and p21 expression in human melanomas. Immunohistochemistry staining analysis revealed an inverse correlation between the expression of HMGB1 and p21 (Figure 6A and Table 3). In line with a p53-indendent mechanism of regulation, there was no significant association between p53 and p21 expression (Table 3), consistent with the results obtained from in vitro studies.

**Table 3:** χ2 test to assess the association between HMGB1, p53 and p21

Markers		HMGB1 staining, n (%)			p value
		Low	High	Total	
p21	Low	10 (31.25%)	22 (68.75%)	32	0.008
	High	29 (61.7%)	18 (38.3%)	47	
p53	negative	18(64.3%)	10(35.7%)	28	0.053
	positive	23(41.8%)	32(58.2%)	55	

### Effect of HMGB1 inhibition on tumorigenicity of melanoma cell lines

We finally conducted studies with mice to validate the HMGB1 deficiency-induced p21-dependent cell growth inhibition for the in vivo relevance. Human melanoma xenograft mouse model was created by implanting the melanoma cells subcutaneously to nude mice. We measured tumor size to monitor the development of melanoma. As expected, melanoma cells with control shRNA (sh-c) developed tumors and the tumor size increased as a function of time. A crucial role for HMGB1 in melanoma development was demonstrated by the finding that depletion of HMGB1 expression resulted in considerable suppression of melanoma tumor development. When compared with sh-c expressing cells, sh-1 and sh-2 expressing melanoma cells exhibited significantly compromised ability to develop tumors as reflected by delayed formation of detectable tumor mass and smaller tumor size (Figure 6B and 6C). Immunohistochemistry analysis of the tumor sections showed an inverse correlation of HMGB1 and p21 expression in that HMGB1-knockdown melanoma tumors expressed elevated level of p21 (Figure 6D). Consistent with a crucial role of HMGB1 in promoting melanoma cell proliferation, a positive correlation between HMGB1 expression and cell proliferation was observed. There were significantly higher staining of proliferation markers Ki-67 and PCNA in HMGB1-proficient melanoma tumors than HMGB1-deficient tumors (Figure 6E). The melanoma xenograft data together provided in vivo evidence indicating a crucial role of HMGB1 in promoting melanoma cell proliferation and progression.

## DISCUSSION

In agreement with the oncogenic role of HMGB1, we found a close association of HMGB1 with human melanoma in that the expression of HMGB1 increased progressively during the development of human melanoma. In support of a crucial role of HMGB1 in melanoma cell proliferation, we demonstrated that decreased expression of HMGB1 via RNAi-mediated knockdown resulted in considerably reduced rate of cell proliferation. The results implicate that human melanoma cells depend on the expression of HMGB1 for the ability to proliferate. In line with this notion was the finding that melanoma cells underwent cell cycle arrest and senescence upon depletion of HMGB1 expression. While further studies are necessary to investigate the mechanism by which melanoma cells depend on HMGB1 for their proliferation, we showed that HMGB1 knockdown was associated with a marked induction of p21, which appeared to be responsible for the observed cell cycle arrest and senescence as these phenotypes were rescued upon depletion the expression of p21. Of interest is the observation that HMGB1 deficiency-induced p21 expression was p53-independent. Indeed, we found that HMGB1 regulated the p21 expression via interacting with Sp1, a transcription factor known for transcriptional regulation of p21. The mechanistic data are consistent with a role of nuclear HMGB1, in line with our observation of its being chiefly nuclear localized in human melanoma cells. HMGB1 contains 2 nuclear localization sequences that guide the protein distribution to the nucleus [1, 37]. Nuclear HMGB1 binds to DNA structure without any sequence specificity and has been shown to function as a DNA chaperone regulating nuclear homeostasis [1, 2, 38]. In addition, HMGB1 has also been reported to interact with transcription factors such as Rb, p73 and the estrogen receptor [39-43] and to modulate the transcription activity. Our results that HMGB1 depletion was associated with induction of Sp1-dependent p21 expression implicate a novel negative regulation of Sp1-mediated transcription.

In summary, we uncovered that HMGB1 was highly overexpressed in melanoma samples when compared with normal skin and nevi tissues. Moreover, the increase of HMGB1 expression correlated with the progression of melanoma and with poorer melanoma patient survival. We show that HMGB1 appeared to be required in support of melanoma cell proliferation because reduced HMGB1 expression caused marked cell cycle arrest and senescence. Consistent with a nuclear function of HMGB1, we demonstrated that HMGB1 regulated cell proliferation via interacting with Sp1 and interfering Sp1-mediated transcription of p21 Together, our study reveals a novel oncogenic role of HMGB1 in promoting human melanoma cell proliferation.

## METHODS

### Cell Culture and Transfection

The human malignant melanoma cell lines, A375 (maintained in our laboratory), G361, SK-MEL-28 and the p53−/− H1299 human lung cancer cells (American Type Culture Collection, Manassas, VA, USA) were used in this study. A375, G361, and p53−/− H1299 cells were cultured in RPMI-1640 (Thermo Scientific, MA, USA) supplemented with 10% FCS (Thermo Scientific). SK-MEL-28 cells and 293FT cells (Clontech Laboratories Inc., CA, USA) were maintained in high glucose DMEM (Thermo Scientific, MA, USA) supplemented with 10% FCS (Thermo Scientific). For transfection experiments, cells were seeded and cultured for 12 h to allow for attachment before transfection. Then, siRNA or plasmids were introduced into cells using TurboFect Transfection Reagent (Thermo Scientific, MA, USA) according to the manufacturer's instructions.

### shRNA or siRNA-mediated Gene Suppression

Three independent lentiviral short hairpin RNA (shRNA) constructs containing a puromycin selection marker were used to target the human HMGB1 gene (sh-1, sh-2, and sh-3; purchased from GeneChem, Shanghai, China). A nonsilencing shRNA (sh-c) was used as control (GeneChem). Three small interfering RNAs (siRNAs) (synthesized by GenePharma, Shanghai, China) were used to target human p21 [44, 45], p53, and Sp1 [46, 47]. A nonsilencing siRNA (si-c) was used as control (GenePharma). The sh-RNA and si-RNA sequences are provided in Supplementary Table S1.

### Western Blot Analyses

Cells were lysed in RIPA buffer and protein concentrations were determined using a BCA Protein Assay kit (Santa Cruz Biotechnology, Santa Cruz, CA). Equal protein was assayed by immunoblot using anti-rabbit HMGB1 (1:1000, Abcam, Cambridge, UK), anti-rabbit p21 (1:200, Santa Cruz Biotechnology, Santa Cruz, CA), anti-mouse p53 (1:1000, Cell Signaling Technology, Danvers, MA), anti-rabbit Sp1 (1:200, Santa Cruz Biotechnology, Santa Cruz, CA), or anti-β-actin (1:10000, Sigma-Aldrich Corp., St. Louis, MO) antibodies followed by peroxidase (HRP)-conjugated goat anti-rabbit or anti-mouse IgG (1:5000, Sigma-Aldrich Corp., St. Louis, MO).

### PCR and Cloning

Total RNA was isolated using TRIzol (Invitrogen, Shanghai, China) according to the manufacturer's instructions. cDNA was synthesized from 1.5 μg RNA using a RevertAid First Strand cDNA Synthesis Kit (Thermo Scientific, MA, USA). Polymerase chain reaction (PCR) was performed. The PCR primers were as follows: Human HMGB1 (hHMGB1) F: GCGAATTCTGGGCAAAGGAGATCCTAAGA, R: GCGGTACCCGCTAGAACCAACTTATTCATCATC; Mouse HMGB1 (msHMGB1) F: CGGAATTCTGGGCAAAGGAGATCCTAAA, R: GGGGTACCACTTATTCATCATCATCATC. After purification using the cycle-pure kit (Sangon Inc. Shanghai, China), both fragments were subjected to restriction enzyme digestion with EcoRI and KpnI (Takara Bio, Otsu, Japan), Myc-pCMV plasmids (Promega, WI, USA) were double digested with the same enzymes, and both the recovered fragments and vectors were incubated with T4 ligase (Takara Bio, Otsu, Japan) at 16°C overnight. The ligation product was transformed and competent bacteria were plated onto the selection medium containing ampicillin. Positive colonies were identified by PCR and sequencing.

### Real-time PCR Analysis

Real-time PCR was performed using the SYBR Green PCR Master Mix kit (Takara Bio, Dalian, China) according to the manufacturer's instructions. Samples were run in a 7500 Fast Real-time PCR System (Applied Biosystems). GAPDH was used as a housekeeping gene. The real-time PCR primers are listed in Supplementary Table S2.

### Proliferation Assays

Proliferation was examined by cell counting and colony formation assays. For cell counting, A375, G361, and SK-MEL-28 cells were collected and counted using a TC10TM Automated Cell Counter (Bio-Rad, USA). For colony formation assays, A375, G361, and SK-MEL-28 cells were transfected with sh-1, sh-2, or sh-c, and seeded in a 6-well plate at a density of 10^4^ cells per well, before being selected with 0.5 μg/ml of puromycin (Sigma-Aldrich Corp., St. Louis, MO). After selection, cells were seeded in 35-mm culture dishes. The cells were cultured for 2 weeks. The dishes were photographed, and the total colony numbers and types of colonies in the plates were evaluated under a microscope. Colonies with > 50 cells were scored. Colony formation efficiency was determined by counting the puromycin-resistant colonies stained with crystal violet solution.

### MTT Assays

MTT [3-(4, 5-dimethylthiazol-2-yl)-2, 5-diphenyltetrazolium bromide] (Sigma, St. Louis, MO) was used for cell viability analysis. Prior to the addition of MTT, A375 cells were transfected with HMGB1 shRNA(sh-2) or/and mouse a HMGB1 expression plasmids (msHMGB1) for 60 h. The prepared cells were seeded in 96-well plates to allow attachment and incubated for another 24 h, 48 h and 72 h. MTT solution 20 μl (5 mg/ml) was added to each well at the correct time and incubated at 37°C for 4 h. After discarding the medium, 150μl DMSO (Sigma, St. Louis, MO) was added to dissolve the formazan crystals. Absorbance was determined using Beckman Coulter-DTX 800 at 595nm.

### Cell Cycle Analysis

Cell cycle staining was conducted using an APC BrdU Flow Kit (BD Biosciences, USA) according to the manufacturer's instructions. Cells were then processed for flow cytometry analysis (BD FACScan). In parallel, cells were stained for cell cycle analysis with propidium iodide (PI). Flow cytometry analysis was then performed (Beckman Coulter, MoFlo™ XDP).

### Senescence-Associated β-Galactosidase (β-Gal) Staining

SA-β-gal activity was evaluated as described by Dimri et al. In brief, A375, G361, and SK-MEL-28 cells transfected with sh-c or sh-2 were fixed by incubation in 3% formaldehyde/0.2% glutaraldehyde/PBS for 5 minutes at room temperature and stained for senescence-associated β-galactosidase activity with X-gal solution containing 1 mg/ml X-gal (Sigma-Aldrich Corp., St. Louis, MO), 40 mM citric acid/sodium phosphate (pH 6.0), 5 mM potassium ferricyanide, 5 mM potassium ferrocyanide, 150 mM NaCl, and 2 mM MgCl_2_ at 37°C overnight.

### Mithramycin A Treatment

A375, G361, and SK-MEL-28 cells were treated with 200 nM mithramycin A (Sigma-Aldrich Corp., St. Louis, MO) for 20 h [34] to inhibit Sp1 before infection with HMGB1 shRNA-expressing viruses. Mithramycin was again added to the cells during selection. Subsequently, the expression levels of HMGB1, Sp1, and p21 were measured by western blot

### Plasmid constructs and Luciferase Assays

The pWWP-Luc [16] was subcloned into the luciferase reporter vector pGL3-Basic (Promega, WI, USA). The pWWP-p53 mut1 vector [49] was a kind gift from Dr. WG Zhu (Department of Biochemistry and Molecular Biology, Peking University Health Science Center, China). The pWWP-p53 mut2 vector was constructed (Invitrogen, Shanghai, China) and subcloned into the pGL3-Basic [49]. In the pWWP-Sp1 mut-Luc plasmid, six mutated Sp1 binding sites [50] were constructed (Invitrogen), and subcloned into the pGL3-Basic. The PG13-Luc and the Sp1-RE-Luc were constructed according to previous literatures [21, 48]. A vacant vector, pGL3-Basic, was used as a control reporter plasmid (Promega, WI, USA). The luciferase constructs are presented schematically in Figure 5C, 5D, and Supplementary Figure S2.

For luciferase assays, A375 and SK-MEL-28 cells stably expressing scrambled (sh-c) or shRNAs targeting HMGB1 (sh-2) were transiently transfected with a vacant pGL3-Basic vector, p21 promoter constructs (wild-type or mutant), PG13-Luc, or Sp1-RE-Luc vector. A Renilla luciferase plasmid was also co-transfected in each experiment as an internal control for transfection efficiency. Cells were harvested 24 or 48 h later. The firefly and renilla luciferase activities were assayed using a Dual-Luciferase Reporter Assay System kit (Promega, Madison, WI, USA). The Firefly luciferase activity was adjusted using the Renilla luciferase activity (Firefly/Renilla ratio). The results are expressed relative to basal reporter (pGL3) activity (set at 1.0).

### Chromatin immunoprecipitation assay (ChIP)

A total number of 2×10^7^ prepared cells were treated with 0.27ml of 37% formaldehyde per 10ml of culture medium with gentle shaking for 10min. Then added 1/10 volume of 1.25M Glycine for 5min to stop the reaction and rinsed the cells with cold PBS. Then cells were washed, lysed and sonicated for 15min at 4°C (20sec on, 20sec off, total 15min) to shear DNA. Collected the supernatant and calculated the protein concentration with Bradford assay. Samples were precleared with Protein A or protein G agarose/salmon sperm DNA beads (Millipore), together with rabbit IgG. Added cell lysis buffer to a total volume 1000ul and rotated at 4°C for 1 hour. Took the supernatants and added HMGB1 antibodies (Abcam, Cambridge, UK) or rabbit IgG for a gentle rotation at 4°C overnight. Then added pre-treated Protein A or protein G agarose/salmon sperm DNA beads into the mixtures, following with an incubation with gentle rotation at 4°C for a hour. The bead-chromatin complexes were spun down with bench centrifugation and washed with Low-salt Solution, High-salt Solution, LiCl_2_ solution, 1×TE Solution sequentially by using a magnetic separation sack. Cross-links were reversed by incubating samples in 10% Chelex-100 (Bio-Rad) for 10min at 99°C. Finally, took the supernatants for PCR.

ChIP DNA was subjected to PCR using specific primers flanking the DNA-binding site for Sp1. The amplified promoter region located at nucleotides from −180 to −29, and the primer sequences were: 5'-TGGAACTCGGCCAGGCTCA-3' (forward); 5'-CAGCGCGGCCCTGATATACA-3' (reverse).

### Co-Immunoprecipitation

293FT cells were transfected with the Myc-pCMV-hHmgb1 and pCMV-Sp1-Flag plasmids (purchased from GeneChem, Shanghai, China). The cells were harvested 36 h later and incubated in IP lysis buffer on ice for 30 min. The protein concentration of each samples was determined using the BCA protein assay (Santa Cruz Biotech, Santa Cruz, CA, USA). Cell lysates were centrifuged at 6000 rpm for 4 min to pellet cellular debris. The supernatant were then incubated with anti-Flag (Sigma-Aldrich Corp., St. Louis, MO), anti-Myc (Santa Cruz Biotech, Santa Cruz, CA, USA) or control IgG at 4°C for 2 hours. Protein G PLUS-agarose beads were then added (Santa Cruz Biotech, Santa Cruz, CA), and the mixtures were rotated overnight at 4°C. After washing thoroughly, the beads were incubated at 95°C for 5min in approximately 40 μl lysis buffer plus 10 μl 5 × loading buffer. The protein samples were subjected to western blot analysis.

### Xenografts Mouse Model

The methods used in this section have been described by our laboratory [51]. Briefly, xenografts were developed using 4- to 6-week-old BALB/c nude mice (Shanghai SLAC Laboratory Animal Co. Ltd., Shanghai, China). Tumor cells were harvested with trypsin-EDTA, washed with RPMI-1640, resuspended in serum-free RPMI-1640, and s.c. inoculated (5 ×10^6^/0.2 ml) into the right axillary fossa. The size of the transplanted tumors was measured every 2 days, and the tumor volume was calculated using the formula Vmm^3^= 1/2 (Length×Width^2^). Animals were housed in a clean vivarium and fed standard mouse chow. The mice were sacrificed 35 days post-inoculation. Harvested tissues were fixed in 10% buffered formalin, embedded in paraffin, sectioned at 5 μm, and stained with H&E. All protocols involving animals were reviewed and approved by the ethical review committee of Central South University of China.

### Patients and Specimens

From January 2000 to January 2007, 136 cutaneous melanomas were diagnosed before treatment at the Department of Pathology as well as the Department of Dermatology, Xiangya Hospital, Central South University. Two pathologists who were both blinded to the patients' clinical data examined tumor slides of the present melanoma cases. Information regarding the patients' gender, age, and tumor sites was available for all cases. After elimination of samples because of heavy pigment, insufficient tumor material, and technical artifacts, 102 samples were available for study. Clinicopathological data are summarized in Table 1. Complete clinical pathological information, including patients' gender, age, tumor thickness (Breslow), ulceration, American Joint Committee on Cancer (AJCC) stage, histological subtype, mitotic index, tumor sites, lymph node metastasis, and distant metastasis, was available for 93 cases. Complete information on patient follow up and time and cause of death was available in 70 cases. There were no significant differences in patients' gender, age, and tumor sites between the four case groups (all p > 0.05). Seventeen cases of normal skin tissues and fifteen cases of normal nevi were included as controls. Informed consent was obtained from all participants. The use of human skin tissues was approved by the medical ethical committee of Xiangya hospital and was conducted in accordance with the Declaration of Helsinki guidelines.

### Immunohistochemical Staining

The sections were dewaxed in turpentine oil and graded alcohols, hydrated, and washed in phosphate-buffered saline. Endogenous peroxidase was inhibited by 3% H_2_O_2_ for 10 minutes (for the HMGB1, p21, and p53 staining) or for 7 minutes (for the Ki-67 and PCNA staining). The sections were pretreated in a microwave oven (in sodium citrate buffer; 0.01 M, pH = 6.0) for 2 minutes (for the p21, Ki-67, and PCNA staining) or 4 minutes (for the HMGB1 and p53 staining) and then incubated with 10% normal goat serum for 30 minutes (for the HMGB1 and p53 staining) or 60 minutes (for the p21 staining). Samples were incubated at 4°C overnight with a primary polyclonal rabbit anti-HMGB1 antibody (1:400, Abcam, Cambridge, UK), a polyclonal rabbit anti-p21 antibody (1:400, Santa Cruz Biotechnology, Santa Cruz, CA), or a mouse anti-p53 antibody (1:200, BioGenex, USA). After three washes, 5 min each with PBS, the sections were incubated with a biotinylated goat anti-rabbit or anti-mouse secondary antibody (Santa Cruz Biotechnology, Santa Cruz, CA) for 30 min. After horseradish peroxidase-streptavidin (Santa Cruz Biotechnology, Santa Cruz, CA) was added to the sections, the samples were stained for 5 min with a 0.05% 3, 3'-diaminobenzidine substrate and then counterstained for 5 min with hematoxylin. Slides were sealed with coverslips and analyzed. Negative controls were treated identically but with the primary antibodies omitted. Formalin-fixed paraffin-embedded blocks from the mouse model were collected in this study, and immunohistochemistry using anti Ki-67 and PCNA antibodies (ZSGB-BIO, Beijing, China) was performed according to the manufacturer's direcitions, using anti HMGB1 and p21 as previously mentioned.

### Evaluation of Immunohistochemical Findings

To analyze the expression of HMGB1, p21, and p53 staining, photos were collected using a Leica DM5000B microscopy equipped with a Leica DFC310FX CCD camera (Leica Microsystems, Germany). Photos of five representative fields were captured using the Leica LAS software (v3.7 Switzerland). Identical settings were used for each photograph, and a uniform setting for all slides was applied. Because HMGB1 expression was diffused in the tumor area, color intensity was presented as the color intensity per area (Integrated optical density, IOD) and evaluated according to the previous literature [52, 53] using the Image-Pro Plus v6.0.0 software (Media Cybernetics Inc, Bethesda, MD). The cutoff for the definition of subgroups was the median value. For immunohistochemical staining of p21, a positive nuclear area above 10% was considered overexpression, and an area below 10% was considered low expression. For p53 staining, both the staining intensity [54] and the proportion of positive tumor cells were recorded. Cases with moderate or strong staining and with more of 10% of positive cells were defined as positive; all others were defined as negative [55]. For Ki-67 and PCNA staining, the percentage of immunoreactive tumor cell nuclei was calculated by counting at least 500 cells within the areas of the most intense nuclear staining.

### Statistical Analysis

All quantified data represent an average of at least triplicate samples unless otherwise indicated. Two-sided p values less than 0.05 were considered statistically significant. The χ^2^ test or Fisher's exact test, Student t test, one-way ANOVA, and Kruskal-Wallis Test were used to determine statistical significance. The associations were also evaluated using Spearman rank correlation methods and/or the χ2 test. Kaplan-Meier analysis (log-rank test) was used to determine the survival. Cox regression model was used for multivariate analysis. Analysis was performed using SPSS 15.0 for Windows (IBM SPSS, Chicago).

## SUPPLEMENTARY FIGURES AND TABLES


