# Supplementary material for: Overexpression of HMGB1 in melanoma predicts patient survival and suppression of HMGB1 induces cell cycle arrest and senescence in association with p21 (Waf1/Cip1) up-regulation via a p53-independent, Sp1-dependent pathway
Source: Oncotarget. 2014 Jul 23;5(15):6387–403. doi: 10.18632/oncotarget.2201 (PMC4171638; doi:10.18632/oncotarget.2201)
Supplement: Supplementary file 1 [file oncotarget-05-6387-s001.pdf]

SUPPLEMENTARY FIGURES AND TABLES

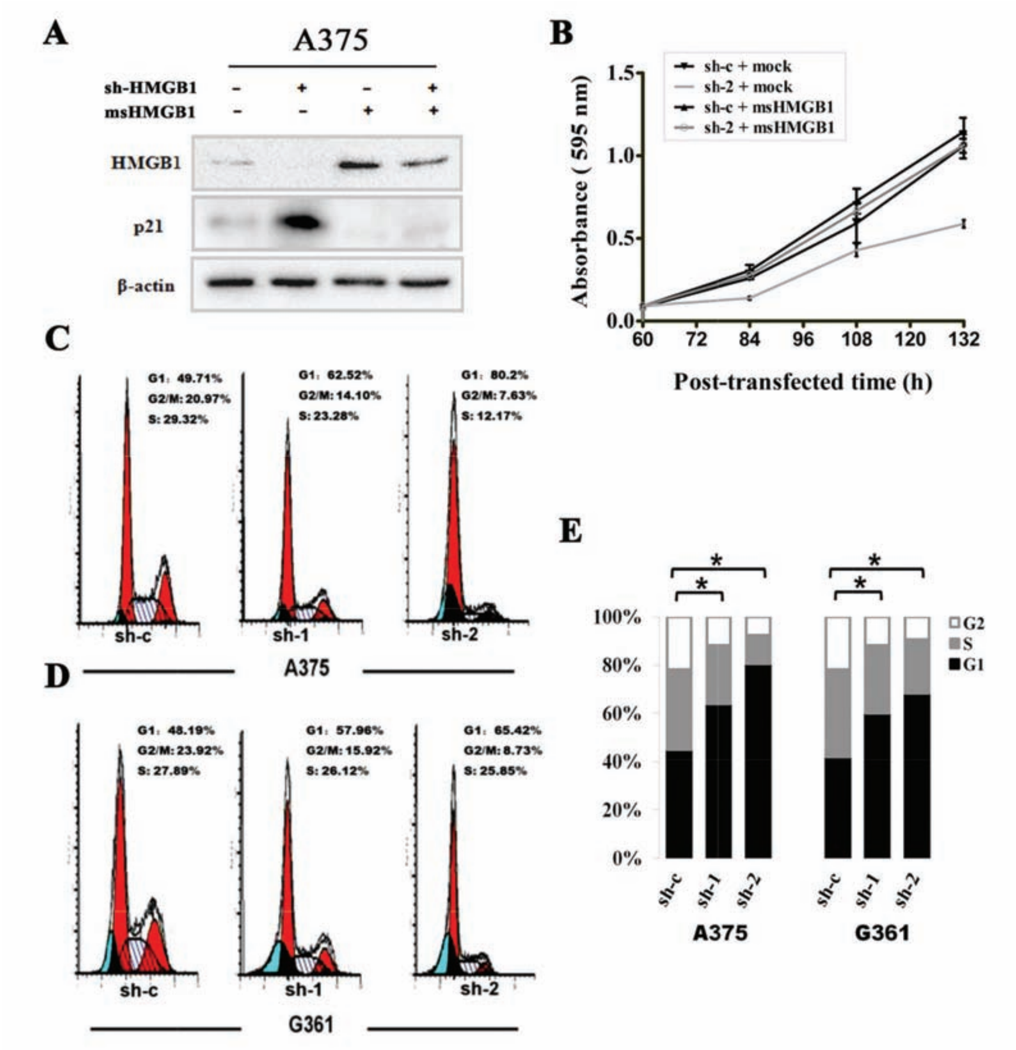

Supplementary Figure S1: Rescue experiment in A375 cells and HMGB1 depletion in A375 and G361 cells induced cell cycle arrest. (A) Re-expression of HMGB1 in shRNAi-HMGB1 expressing A375 cells as shown by western blots. The upregulated protein levels of p21 in shRNAi-HMGB1 expressing A375 cells were decreased upon on the reintroduction of HMGB1 using a mouse HMGB1 expression plasmid (msHMGB1). **(B)** As shown by MTT assay, the proliferation of cells in which HMGB1 knocked down was suppressed, and the re-expression of HMGB1 completely rescued the retarded rate of cell proliferation in the shRNAi expressing A375 cells. mock, control pCMV plasmid. (C-D) Representative Cell Cycle profiles of HMGB1 depleted (sh-1 and sh-2) or control (sh-c) in A375 **(C)** and G361 **(D)** by staining with propidium iodide (PI) using FACS analysis. **(E)** The numbers of cells in each cell cycle were quantified and presented as mean ± SD from three independent experiments. Bars, SD; *P <0.05

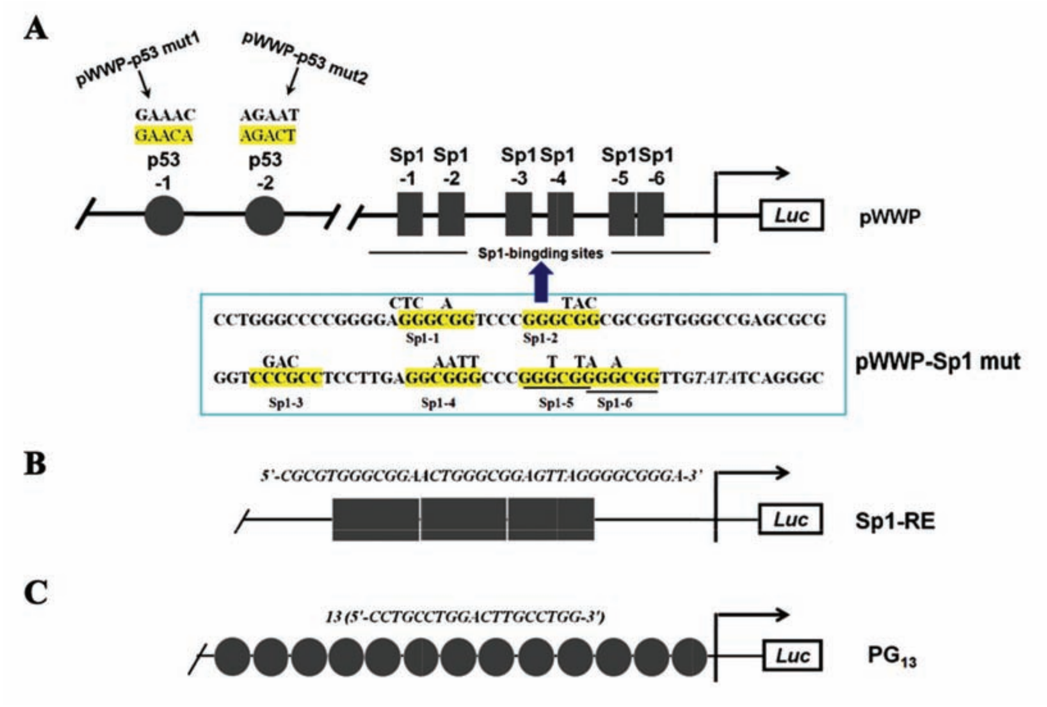

Supplementary Figure S2: Schematic presentation of luciferase constructs. **(A)** Full-length (pWWP-Luc), mutant p21 promoter luciferase plasmids (pWWP-p53 mut1, pWWP-p53 mut2, and pWWP-Sp1 mut). The human wild-type p21 promoter luciferase fusion plasmid (full-length), pWWP-Luc promoter construct, was made from a 2.4-kb genomic fragment of p21 promoter containing the transcriptional start site, two p53 binding sites and six Sp1-binding sites. The pWWP-p53 mut1 vector, in which the first p53-binding site, GAACA (−2234 to −2230 relative to the translational start site) was replaced with GAAAC in pWWP-p53 mut1-Luc. The pWWP-p53 mut2 vector, in which the second binding site, AGACT (−1344 to −1340 relative to the translational start site) was replaced with AGAAT in pWWP-p53 mut2-Luc. The pWWP–Sp1 mut luciferase reporter plasmid contains six mutated Sp1 binding sites. Sequences shown in reverse color show consensus binding sites for the transcription factor Sp1 (sites 1–6). Nucleotide substitutions that were introduced into the p21 promoter are shown on top of the sequence. **(B)** The Sp1 responsive elements (Sp1-RE-Luc, which contains three consensus Sp1 binding sites, and **(C)** PG13-Luc, which contains thirteen consensus p53 binding sites (5'-CCTGCCTGGACTTGCCTGG-3'), driving luciferase plasmids used in transfection assays.

Supplementary Table S1. Sequences of shRNA/siRNA

**shRNA/ siRNA**		**sequences**
HMGB1 shRNA	sh-1	5’-CcggATTGCTGCATATCGAGCTAAACTCGAGTTTAGCTCGATATGCAGCAATTTTTTg-3’<br>5’-aattcaaaaaATTGCTGCATATCGAGCTAAACTCGAGTTTAGCTCGATATGCAGCAAT-3’
	sh-2	5’-CcggTTGGTGATGTTGCGAAGAAACCTCGAGGTTTCTTCGCAACATCACCAATTTTTg-3’<br>5’-aattcaaaaaTTGGTGATGTTGCGAAGAAACCTCGAGGTTTCTTCGCAACATCACCAA-3’
	sh-3	5’-CcggGATGCAGCTTATACGAAATAACTCGAGTTATTTCGTATAAGCTGCATCTTTTTg-3’<br>5’-aattcaaaaaGATGCAGCTTATACGAAATAACTCGAGTTATTTCGTATAAGCTGCATC-3’
p53 siRNA		5’-UCAAAUCAUCCAUUGCUUGGG-3’<br>5’-CAAGCAAUGGAUGAUUUGAUG-3’
		5’-UGAACAUGAGUUUUUUAUGGC-3’<br>5’-CAUAAAAAACUCAUGUUCAAG-3’
p21 siRNA(44, 45)		5’-CUUCGACUUUGUCACCGAG-3’<br>5’-CAU ACUGGCCUGGACUGUUUU-3’
Sp1 siRNA(46, 47)		5’-GGUAGCUCUAAGUUUUGAUtt-3’<br>5’-GGUCAUUUCUUUGCUUAUGtt-3’<br>5’-UGUAGAGUCUGCCAACUGACCUGUC-3′

HMGB1 shRNAs were purchased from GeneChem company, Ltd, Shanghai, China; p53siRNAs were designed using siDirect (http://design.RNAi.jp/).

Supplementary Table S2. Real time RT-PCR primers

Symbol	Forward primer (5’-3’)	Reverse primer (5’-3’)
HMGB1	TTGCGAAGAAACTGGGAGAG	CAGCCTTGACAACTCCCTTT
p21	AGCAGCGGAACAAGGAGT	CGTTAGTGCCAGGAAAGACA
GAPDH	CGACCACTTTGTCAAGCTCA	AGGGGTCTACATGGCAACTG
p16	ATATGCCTTCCCCCACTACC	CGTGAGTGCTCACTCCAGAA
p18	GGACCCAGGACTATCCCTTC	TTTAGGGTCCCTTGTTCACG
p15	GCGGATTTCCAGGGATATTT	CACCAGGTCCAGTCAAGGAT
p27	CAGGTAGTTTGGGGCAAAAA	ACAGCCCGAAGTGAAAAGAA
p57	AGAGATCAGCGCCTGAGAAG	TGGGCTCTAAATTGGCTCAC

Primers used in PCR were designed using Primer Premier 5.0 (Premier Biosoft International, Palo Alto, CA) and the online utility Primer 3 (http://frodo.wi.mit.edu/primer3/).
